# The relation between Schneiderian membrane thickening and radiodiagnostic features of periapical pathology

**DOI:** 10.1590/0103-6440202405775

**Published:** 2024-08-30

**Authors:** Nicolly Oliveira-Santos, André Ferreira Leite, Eline Petitjean, Andres Torres, Dominique Van der Veken, Frederik Curvers, Jáder Camilo Pinto, Paul Lambrechts, Renhilde Jacobsi

**Affiliations:** 1 OMFS IMPATH Research Group, Department of Imaging & Pathology, University Hospitals Leuven, KU Leuven, Leuven, Belgium.; 2 Department of Oral Health Sciences, University Hospitals Leuven, KU Leuven, Leuven, Belgium; 3 Department of Restorative Dentistry, School of Dentistry, São Paulo State University, Araraquara, São Paulo, Brazil; 4 Department of Oral Health Sciences, University Hospitals Leuven, KU Leuven, Leuven, Belgium.; 5 Department of Dental Medicine (DENTMED), Karolinska Institutet, Stockholm, Sweden

**Keywords:** apical periodontitis, cone-beam computed tomography, maxillary sinus, Schneiderian membrane

## Abstract

This study aimed to assess the relationship between Schneiderian membrane thickening and periapical pathology in a retrospective analysis of Cone Beam Computed Tomography (CBCT) images. For this, 147 CBCT scans containing 258 sinuses and 1,181 teeth were assessed. Discontinuation of the lamina dura, widening of the periodontal ligament space, apical periodontitis (AP), and partly demineralized maxillary sinus floor associated with AP were considered periapical pathology. Maxillary sinus mucosal thickening (MSMT) was classified as odontogenic or non-odontogenic. An irregular band with a focal tooth associated thickening and local thickening related to a root were considered odontogenic types of MSMT. The relation between the imaging features of periapical pathology and the type and thickness of MSMT was determined by logistic regression and linear mixed model, respectively. In addition, linear regression and Mann Whitney test evaluated the relation and demineralization of the AP lesion towards the sinus floor (p≤0.05). The odds of having an odontogenic type of MSMT were significantly higher when a periapical pathology was present in the maxillary sinus. Eighty-two percent of AP partly demineralized towards the sinus floor were associated with an odontogenic MSMT. Both AP lesions partly demineralized towards the sinus floor and, with increased diameter, led to increased MSMT. In conclusion, there is an 82% risk of having an odontogenic type of MSMT with the presence of AP partly demineralized towards the sinus floor. More thickening of the maxillary sinus mucosa is seen with larger AP lesions and partial demineralization of the sinus floor.

## Resumo

O objetivo deste estudo foi avaliar a relação entre o espessamento da membrana Schneideriana e periapicopatia em uma análise retrospectiva de imagens de Tomografia Computadorizada de Feixe Cônico (TCFC). Para isso, foram avaliadas 147 imagens de TCFC, contendo 258 seios maxilares e 1.181 dentes. A descontinuação da lâmina dura, o aumento do espaço do ligamento periodontal, a periodontite apical (PA) e o assoalho do seio maxilar parcialmente desmineralizado associado à PA foram considerados sinais de periapicopatia. O espessamento da mucosa do seio maxilar foi classificado como odontogênico ou não-odontogênico. Uma faixa irregular com um dente focal associado ao espessamento e espessamento local em relação a uma raiz foram considerados como tipos odontogênicos de espessamento da mucosa do seio maxilar. A relação entre a periapicopatia e o tipo de espessamento da mucosa do seio maxilar foi determinada (p≤0.05). A probabilidade de se ter um tipo odontogênico de espessamento da mucosa do seio maxilar foram significativamente maiores quando uma periapicopatia estava presente no seio maxilar. Oitenta e dois por cento da PA parcialmente desmineralizada em direção ao assoalho do seio estavam associados a um espessamento odontogênico da mucosa do seio maxilar. Ambas as lesões de PA parcialmente desmineralizadas em direção ao assoalho do seio e com aumento do diâmetro levaram a um aumento do espessamento da mucosa do seio maxilar. Em conclusão, com a presença de periapicopatia, há um risco maior de ter um tipo odontogênico de espessamento da mucosa do seio maxilar. Um espessamento maior da mucosa do seio maxilar é observado com lesões de PA maiores e desmineralização parcial do assoalho do seio maxilar.

## Introduction

A close relationship between the maxillary sinus floor and roots of the posterior maxillary teeth (mainly) brings this anatomical structure into the diagnostic domain of the dentist. Distances between the apices of those teeth and the maxillary sinus floor vary individually; however, molars have a greater likelihood of proximity than premolars. The apex of the mesiobuccal root of the second maxillary molar has been described as the closest to the maxillary sinus floor, while the apex of the buccal root of the first premolar has been the furthest away. Occasionally, the anterior border of the maxillary sinus spreads to the canine level ^1^.

Considering the anatomical proximity, dental pathology can spread to the maxillary sinus. Even if the cortical sinus floor is intact, odontogenic inflammatory products can be transported directly through the maxillary bone marrow or via blood vessels and lymphatics toward the maxillary sinus ^2^. Conditions like apical periodontitis (AP), advanced periodontal disease, dentoalveolar trauma, oro-antral communications following dentoalveolar surgery, dental implant placement, sinus floor elevation procedures, and intrusion of foreign materials into the maxillary sinus can harm the integrity of the Schneiderian membrane, the inner mucosal lining of the maxillary sinus cavity. It can cause an inflammatory reaction with hyperplasia of the Schneiderian membrane, known as mucosal thickening, which may indicate odontogenic maxillary sinusitis ^3^. Therefore, the detection of mucosal thickening in the maxillary sinus may characterize the detection of odontogenic maxillary sinusitis.

Cone-beam computed tomography (CBCT) image is a valuable imaging modality for detecting odontogenic maxillary sinusitis ^(^ ^4^. Prevalence studies based on CBCT have detected mucosal thickening of >2 mm in 36 to 60.5% of the maxillary sinuses ^5^ ^,^ ^6^ ^,^ ^7^ ^,^ ^8^ ^,^ ^9^. These mucosal changes are frequently asymptomatic in most patients, even though thickening may be related to a certain degree of irritation, being either of rhinogenic or odontogenic origin ^10^.

Previous studies have shown a higher probability of maxillary sinus mucosal thickening when attached to teeth presenting AP or severe periodontal bone loss ^5^ ^,^ ^6^ ^,^ ^11^ ^,^ ^12^ ^,^ ^13^ ^,^ ^14^, especially when molar roots with AP are closer to the maxillary sinus ^15^. These results indicate the association between AP and maxillary sinus mucosal thickening. Nevertheless, some imaging characteristics of the periapical lesions, such as the corticalization of the AP lesion, have not been studied, and their association with mucosal thickening has not yet been unraveled.

The aim of this study was first to assess the relationship between periapical pathology, such as root infection and AP lesion, and the Schneiderian membrane thickening. Secondly, to evaluate the influence of the imaging features of periapical pathology, especially AP lesions, on the thickness and type of maxillary sinus mucosal thickening.

## Materials and methods

### Study population

Three hundred eighty CBCT scans of patients referred to the Dentomaxillofacial Radiology Center of the University Hospital of Leuven were assessed. All CBCT scans were taken for different diagnostic reasons, such as implant planning, orthognathic surgery, and assessment of maxillofacial and endodontic pathosis. No patients underwent CBCT scans exclusively for this study. Scans were included when the entire maxillary floor of at least one maxillary sinus was visible. Only maxillary canines, premolars, and molars were assessed, and these teeth and their periapical region had to be completely visible on the volume to be included. CBCT scans were excluded when showing extensive motion or metal artefacts or if containing no teeth, primary teeth, permanent teeth with open apices, teeth with periodontal pathology, maxillary implants, oroantral communications as well scans taken after sinus augmentations, maxillofacial trauma, and orthognathic surgery. Impacted teeth were also excluded from the analysis. A final set of 147 scans from 59 males and 88 females (mean age 46.6 ± 16.3 years, range 15-84 years) containing 258 sinuses and 1 181 teeth were available for assessment.

### Imaging evaluation

All CBCT scans were taken using the 3D Accuitomo 170 device (3D Accuitomo, J. Morita, Kyoto, Japan), with 3-5 mA, 90 kV, and 0.16-0.25 voxel size. Scans were evaluated using the i-Dixel 2.0 software (J. Morita USA Inc., Irvine, CA, USA) on a 30” monitor with a resolution of 2560 × 1600 pixels (Dell 3008WFP, Dell Inc., Round Rock, TX, USA) in a dark room. Two observers, senior postgraduate students in endodontics, screened all CBCT scans independently. Before the examination, 20 CBCT scans not included in this study were screened for calibration purposes. In the event of discrepancies between the observers, both analyzed the images together to reach a consensus. Each scan was analyzed in all its planes for the following parameters:

### Assessment of the maxillary sinus

**Figure 1:**
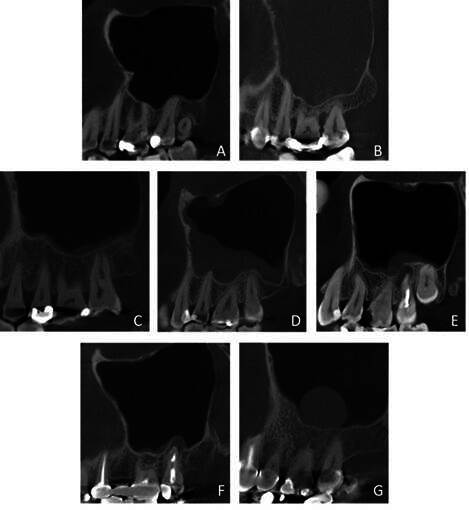
Type of maxillary sinus mucosal thickening. (A) Invisible thickening. (B) Full opacification. (C) Regular band. (D) Irregular band of non-odontogenic origin. (E) Irregular band with focal tooth-associated thickening. (F) Local thickening related to a root. (G) Local thickening unrelated to a root.

**Figure 2:**
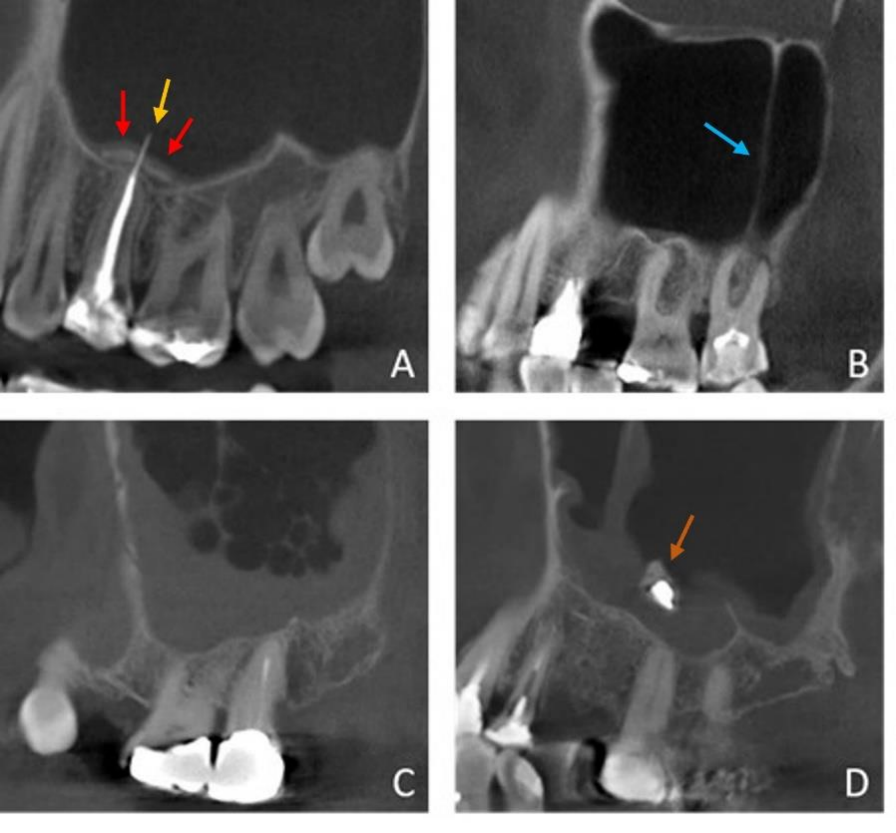
Findings of the maxillary sinus. (A) Calcifications (red arrows) as a reaction to the overextension of gutta-percha (yellow arrow) in tooth 25 that penetrates the maxillary sinus. (B) Septum of the maxillary sinus (blue arrow). (C) Air bubbles associated with an inflammatory maxillary sinus reaction. (D) Foreign body (orange arrow) associated with maxillary sinus mucosal thickening.

Thickening of the maxillary sinus mucosa was measured in the long-axis of each tooth perpendicular to the maxillary sinus floor using the i-Dixel software’s measuring tool. Considering that mucosal thickening may be of rhinogenic or odontogenic origin, the mucosal thickening was divided into different types. The type of thickening was classified per sinus as invisible thickening (<2 mm), regular band, irregular band of non-odontogenic origin, irregular band with focal tooth associated thickening, local thickening in relation with a root, local thickening on the maxillary sinus floor but not in relation with a root and full opacification (Figure 1). A band was considered to cover the entire maxillary sinus floor; a local thickening was restricted up to two adjacent teeth. An irregular sinus band related to a focal tooth thickening and a local sinus thickening associated with a tooth root were considered odontogenic types of maxillary sinus mucosal thickening. Calcification reaction associated with mucosal thickening, foreign body of non or odontogenic origin, air bubbles of acute inflammatory reaction, and maxillary sinus floor associated septa with a minimum height of 2.5 mm were noted (Figure 2).

### Assessment of dental conditions

**Figure 3:**
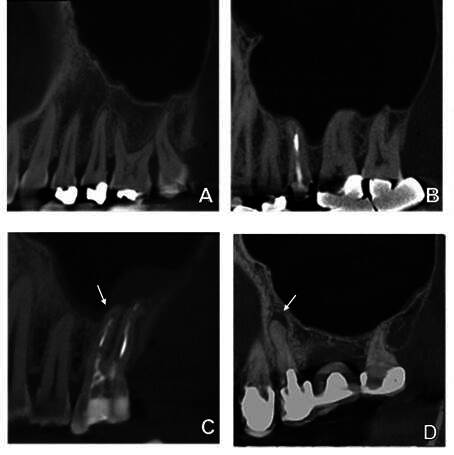
Periradicular conditions. (A) All teeth have an intact lamina dura. (B) The lamina dura of all teeth is fused with the maxillary sinus floor in their periapical region. (C) Discontinuation of the lamina dura on the mesiobuccal root of tooth 26. (D) Apical periodontitis on tooth 25.

The lamina dura (LD), being the fine radiopaque inner lining of the dental alveolus, was classified in the periapical region of each root as intact, fused with the maxillary sinus floor or showing signs of root infection (Figure 3A-C). Discontinuation of the LD or widening of the periodontal ligament space in the periapical region were assumed as signs of root infection.

**Figure 4:**
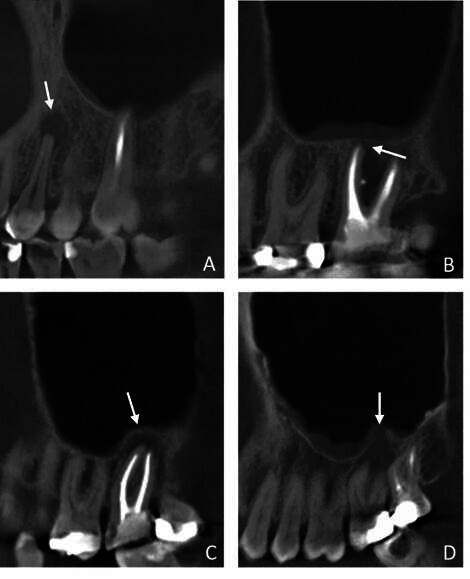
Parameters of the apical periodontitis (AP). (A) The AP on tooth 24 is distant from the maxillary sinus floor, which is fully corticated. (B) The AP on tooth 27 touches the maxillary sinus floor, which is fully corticated. (C) The AP on tooth 27 protrudes into the maxillary sinus floor, which is fully corticated. (D) The AP on tooth 27 protrudes into the maxillary sinus floor, which is partly demineralized. It is a local osteolytic process - the neighboring structures were unaffected.

Apical periodontitis was defined as a radiolucency in connection with the apical part of the root, exceeding at least two times the width of the lateral part of the periodontal ligament ^16^ (Figure 3D). AP was measured on a root level. The diameter of the AP was measured using the i-Dixel software’s measuring tool in the axial sections. The spatial relationship between the periapical pathology and the maxillary sinus floor was classified as distant, touching, or protruding (Figure 4). The degree of corticalization of the AP lesion in relation to the maxillary sinus floor was classified as fully corticated or partly demineralized. When the AP involved several roots, the same measurements were used for all roots involved in the AP.

For the value of the diameter of the AP and the thickening of the maxillary sinus mucosa, the average of both observers’ measurements was calculated on the condition that the difference was less than or equal to 2 mm. In case of more than a 2 mm discrepancy, both observers redone the measurement, and the average was calculated.

Statistical methods

Analyses were performed using SAS software, version 9.4 of the SAS System for Windows (of SAS Institute Inc., Cary, NC, USA) and the GraphPad Prisma 7.00 statistical software package (GraphPad Software, La Jolla, CA, USA), with a level of significance of 5%.

A linear mixed model with a random effect on the patient and a random effect on the maxillary sinus (nested within the patient) was used to evaluate the relation between a tooth with at least one root with a periapical pathology and the thickness of the maxillary sinus membrane. Then, a linear regression model was used to evaluate the relation between the AP diameter and mean sinus mucosa thicknesses. Besides, the Mann-Whitney test was used to compare the AP diameter between partly demineralized and fully corticated AP lesions towards the sinus floor. In addition, a logistic regression model evaluated the relation between the presence of at least one root with a periapical pathology and an odontogenic type of maxillary sinus mucosal thickening.

A post hoc power analysis was performed using the software package G*Power (version 3.1.9.2.) based on the condition that showed statistically significant differences, considering the effect size, alpha, sample size, and the number of groups, which resulted in 99%.

## Results

Maxillary sinus mucosal thickening was present in 143 sinuses (55%). The prevalence of the different types of maxillary sinus mucosal thickening is presented in Table 1. Ninety-eight sinuses (38%) were associated with at least one root with root infection, and 57 sinuses (22%) were associated with at least one root with AP. In 16 sinuses (6%), the AP lesion showed a partial demineralization of the maxillary sinus floor. Septa associated with the maxillary sinus floor was present in 80 sinuses (31%). Calcifications, air bubbles, and foreign bodies were detected less frequently in 18 (7%), 7 (3%), and 5 (2%) maxillary sinuses, respectively.

**Table 1:** Prevalence of the types of maxillary sinus mucosal thickening. N: number of maxillary sinuses.

Type of maxillary sinus mucosal thickening	n/N (%)
Invisible thickening	115/258 (44.6%)
Regular band	27/258 (10.5%)
Irregular band of nonodontogenic origin	33/258 (12.8%)
Irregular band with focal tooth associated thickening	25/258 (9.7%)
Local thickening in relation with a root	27/258 (10.5%)
Local thickening on the maxillary sinus floor but not in relation with a root	27/258 (10.5%)
Full opacification	4/258 (1.5%)

One hundred and twenty-one teeth (10%) had at least one root with root infection, and 64 teeth (5%) had at least one root with AP. Eight hundred and seventy-five teeth (74%) were not associated with thickening of the maxillary sinus mucosa, and 306 teeth (26%) were associated with a thickening. The mean thickness of the maxillary sinus membrane was 2.2 ± 4.6 mm.

Ninety-six roots (4%) had AP, and the mean diameter of an AP lesion was 6.0 ± 5.3 mm. The relation between the AP and the maxillary sinus floor was touching for 22 AP lesions (23%), and 45 AP lesions (47%) protruded into the maxillary sinus floor. Thirty-one AP lesions (32%) showed some demineralization with only partial bone demineralization towards the maxillary sinus floor. The presence of a partly corticated sinus floor (partial demineralization) yielded significantly more extensive AP lesions (P<0.05).

### Analysis of maxillary sinus mucosal thickness (tooth level)

The maxillary sinus mucosa was thicker in the long-axis of a tooth with at least one root with root infection (P<0.0001). The mean thickness of the maxillary sinus mucosa in the long-axis of a tooth with and without root infection was 3.2 mm (2.5 - 4.0 95% CI) and 2.1 mm (1.5 - 2.6 95% CI), respectively. Table 2 shows an overview of the results of the linear mixed models evaluating the effect of a tooth with at least one root with a periapical pathology on the maxillary sinus mucosal thickening. The sinus membrane was thicker when a tooth showed at least one root with a periapical pathology present. When comparing the least-squares mean sinus mucosa thicknesses of all the periapical pathologies, only the combination AP with a partial bone demineralization of the sinus floor led to a significantly thicker sinus mucosa than the other periapical pathology conditions (P<0.05). Besides, there was a positive correlation between AP lesion diameter and mean sinus mucosa thickness.

**Table 2:** (Least-squares) mean sinus membrane thickness (mm) (95% CI min-max) as a function of the presence of at least one root in the tooth with a periapical pathology. Results obtained from linear mixed models for each pathology separately, fitted on 1181 teeth. N: number of teeth with at least one root with a periapical pathology (note that these pathologies are not mutually exclusive).

	Root sign			
Description of the periapical pathology	Absent	Present	N	P
Root infection	2.07 (1.49 - 2.65)	3.23 (2.48 - 3.98)	121	<0.0001
Root infection and touching/protruding relation root-sinus floor	2.13 (1.56 - 2.71)	3.94 (2.93 - 4.94)	45	<0.0001
AP	2.13 (1.54 - 2.71)	3.45 (2.57 - 4.33)	64	0.0002
AP and touching/protruding relation AP-sinus floor	2.15 (1.57 - 2.73)	3.78 (2.77 - 4.79)	44	0.0002
AP and partial demineralization sinus floor	2.16 (1.58 - 2.74)	5.24 (3.84 - 6.63)	19	<0.0001

### Analysis of the type of maxillary sinus mucosal thickening (sinus level)

Distribution of the type of maxillary sinus mucosal thickening according to the type of periapical pathology is provided in Table 3. The odontogenic types of maxillary sinus mucosal thickening (an irregular band with a focal tooth associated thickening and local thickening in relation to a root) were the most observed types of maxillary sinus mucosal thickening in relation to periapical pathology. Only 7 out of 160 maxillary sinuses associated with no periapical pathology (4%) showed an odontogenic sinus mucosal thickening. There was an odontogenic type of maxillary sinus mucosal thickening in 13 of the 16 sinuses (82%) associated with at least one root with AP combined with partial bone demineralization towards the maxillary sinus floor. In 87 non-infected sinuses (54%), sinus mucosal thickening was not observed.

**Table 3:** Frequency of the type of maxillary sinus mucosal thickening according to type of periapical pathology. Row percentages were given per type of periapical pathology. The table ignores the differences in the number of included roots per sinus.

Type of periapical pathology	Type of thickening							
**Frequency *(%)***	invisible	regular band	irregular band of nonodontogenic origin	irregular band with focal tooth associated thickening	local thickening in relation with root	local thickening not in relation with root	full opacification	Total
No periapical pathology	87 *(54.4)*	20 *(12.5)*	21 *(13.1)*	0 *(0.0)*	7 *(4.4)*	22 *(13.7)*	3 *(1.9)*	160
Root infection + distant relation root-sinus floor	10 *(47.6)*	1 *(4.8)*	4 *(19.0)*	3 *(14.3)*	2 *(9.5)*	1 *(4.8)*	0 *(0.0)*	21
Root infection + touching/protruding relation root-sinus	5 *(25.0)*	0 *(0.0)*	3 *(15.0)*	6 *(30.0)*	5 *(25.0)*	1 *(5.0)*	0 *(0.0)*	20
AP + distant relation root-sinus floor	6 *(31.6)*	2 *(10.5)*	2 *(10.5)*	1 *(5.3)*	5 *(26.3)*	3 *(15.8)*	0 *(0.0)*	19
AP + touching/protruding relation AP-sinus floor + fully corticated sinus floor	6 *(27.3)*	3 *(13.6)*	2 *(9.1)*	6 *(27.3)*	4 *(18.2)*	0 *(0.0)*	1 *(4.5)*	22
AP + touching/protruding relation AP-sinus floor + partly demineralized sinus floor	1 *(6.2)*	1 *(6.2)*	1 *(6.2)*	9 *(56.2)*	4 *(25.0)*	0 *(0.0)*	0 *(0.0)*	16
Total	115	27	33	25	27	27	4	258

Table 4 provides an overview of the results of the logistic regression models evaluating the effect of at least one root in the sinus with a periapical pathology on the odds of an odontogenic type of maxillary sinus mucosal thickening. For all conditions of periapical pathology, odds ratios of an odontogenic type of maxillary sinus mucosal thickening were significantly higher when at least one root in the sinus with a periapical pathology was present.

**Table 4:** Odds ratios for the presence of an odontogenic type of maxillary sinus mucosal thickening (95% CI min-max) as a function of at least one root in the sinus with a periapical pathology. Results obtained from logistic regression models for each sign separately, fitted on 258 sinuses. N: number of sinuses with at least one root with a periapical pathology (note that these signs are not mutually exclusive).

	Root sign			
Description of the periapical pathology	Absent	Present	N	P
Root infection	4.3 (2.0 - 8.9)	45.5 (34.7 - 56.7)	98	<0.0001
Root infection and touching/protruding relation root-sinus floor	12.1 (8.2 - 17.7)	61.3 (43.2 - 76.8)	40	<0.0001
AP	10.6 (6.8 - 16.1)	52.1 (37.0 - 66.8)	57	<0.0001
AP and touching/protruding relation AP-sinus floor	12.3 (8.3 - 17.9)	62.1 (43.7 - 77.6)	38	<0.0001
AP and partial demineralization sinus floor	15.3 (10.9 - 21.0)	82.5 (54.5 - 94.9)	16	<0.0001

In the subset of patients with one maxillary sinus having at least one root with AP and the other sinus having no AP, 16 of 30 patients (53%) had an odontogenic type of maxillary sinus mucosal thickening in the sinus associated with AP (Table 5). At the same time, neither thickening nor odontogenic mucosal thickening was noted in the contralateral maxillary sinus without AP (Table 5). No patient presented an opposite scenario: an odontogenic type of maxillary sinus mucosal thickening in the sinus without AP or a non-odontogenic type of maxillary sinus thickening in the sinus with AP.

**Table 5:** Comparison of type of maxillary sinus mucosal thickening between a sinus associated with at least one root with AP and a sinus having no roots with AP within the same subject. Paired analysis on patient level.

	Tooth root		
**Frequency *Percent***	Without AP	With AP	Total
Odontogenic thickening %	10 *33.3*	16 *53.3*	26 *86.7*
Non-odontogenic thickening %	0 *0.0*	4 *13.3*	4 *13.3*
Total %	10 *33.3*	20 *66.7*	30 *100.0*

## Discussion

An unhealthy periapical condition of maxillary posterior teeth has previously been associated with a higher prevalence of maxillary sinus mucosal thickening ^5^ ^,^ ^14^ ^,^ ^15^. The present study is, however, the first to show that the imaging features of periapical pathology may also impact the thickness of the mucosal thickening. Indeed, a thicker and odontogenic type of mucosal thickening may be observed in the presence of an AP lesion towards the maxillary sinus floor with partial bone demineralization. This observation should trigger the dentist to treat the tooth and avoid further development of odontogenic sinusitis.

Maillet *et al.* (2011)^17^ modified the classification by Abrahams & Glassberg (1996) ^18^ and defined odontogenic sinusitis as a localized thickening in association with either an extraction site or a tooth with caries, a defective restoration or a periapical lesion. If mucosal thickening was not limited to any tooth, but one of the aforementioned dental pathologies was present, then the sinusitis was of undetermined origin. A problem of this classification system is the difficulty of assessing caries and the quality of coronal restorations on CBCT due to artifacts related to beam hardening ^19^. Our study considered local thickening related to a root and an irregular band with focal tooth-associated thickening as odontogenic types of maxillary sinus mucosal thickening. These were indeed the most observed types of maxillary sinus mucosal thickening in relation to periapical pathology. The fact that the vast majority (96%) of maxillary sinuses without periapical pathology were free from sinus mucosa thickening reinforced the hypothesis that, generally, the odontogenic type of maxillary sinus mucosal thickening was strongly associated with periapical pathology. In addition, all the cases from the 4% maxillary sinuses without periapical pathology but with an odontogenic type of mucosal thickening had at least one tooth protruding to the maxillary sinus floor. Therefore, as Nascimento *et al.* (2016) ^20^ observed, the anatomic relationship between tooth and maxillary sinus floor may also be associated with odontogenic maxillary sinus mucosal thickening.

Endodontic infections and disruption of the Schneiderian membrane may lead to mucosal inflammation and impair mucociliary function within the maxillary sinus ^21^. It explains the thicker mucosal thickening found in the maxillary sinus associated with at least one root with periapical pathology (discontinuation of the LD, widening of the periodontal ligament space in the periapical region, fully corticated AP or partial demineralized AP). Furthermore, the present results suggest that a higher prevalence and thickness of mucosal thickening is also associated with a closer spatial relationship between the periapical pathology and the maxillary sinus floor. Similarly, the AP lesion’s size and cortication are associated with the thickness of the maxillary sinus mucosal thickening.

Lu *et al.* (2012)^12^ graded AP using the periapical index scoring system ^22^ and found a positive correlation between the degree of AP and the prevalence of sinus mucosal thickening. They suggested that increased bacterial load results in more severe AP and thereby increases the likelihood of maxillary sinus mucosal thickening since bacteria and their toxins can infiltrate the maxillary sinus, infecting it ^12^. Nunes *et al.* (2016)^23^ graded AP using the CBCT periapical index ^24^ and observed that when the AP diameter was >8 mm, all maxillary sinuses had thickening of the Schneiderian membrane. Corroborating with the previous literature, in the present study, an increasing diameter of AP led to higher thickness of the maxillary sinus mucosal thickening.

In addition, partial bone demineralization of an AP lesion towards the maxillary sinus floor led to an even higher thickness of maxillary sinus mucosa compared to the other periapical pathology conditions evaluated. Hence, it seems that the integrity of the maxillary sinus floor is a barrier to the spread of periapical infection. While few studies showed that the proximity of the periapical lesion towards the maxillary sinus may increase the risk for mucosal thickening ^15^ ^,^ ^20^, to the authors’ knowledge, the present study is the first to show a link between partial demineralization of the sinus floor around AP lesion and the degree of mucosal thickening of the maxillary sinus floor. In addition, all periapical pathology conditions, but especially AP lesions associated with partial bone demineralization of the sinus floor, were significantly associated with odontogenic types of maxillary sinus mucosal thickening, corroborating our hypothesis of odontogenic sinusitis. However, considering that it was a retrospective study, it is important to highlight that the association found does not mean causation; the presence of a periapical pathology does not necessarily mean the development of a maxillary sinus mucosal thickening.

This study had certain limitations related to the retrospective nature of the study. Indeed, patients were referred for CBCT for different diagnostic reasons. Despite applying exclusion criteria, it does not represent a random population sample. Therefore, extrapolation of results concerning prevalence should be carried out with caution. Secondly, since this was a cross-sectional study, the subset of sinuses associated with periapical pathology was relatively small. A larger sample size might have revealed more significant associations concerning the effect of anatomical proximity of the AP and the tooth type in which the infection was located. Third, only imaging mucosal changes were assessed, and no correlations with clinical symptoms or patients’ sinusitis-related history were made. In this respect, clinical and imaging data correlations might be advised in future studies. Also, to establish a cause-effect relationship between periapical pathology and sinus mucosal thickening, future prospective studies based on large sample size data should be performed to evaluate the effect of dental therapy on the healing modalities of the maxillary sinus membrane.

In conclusion, periapical pathology of maxillary teeth, especially with intimate contact with the maxillary sinus floor, is associated with a higher probability of having odontogenic maxillary sinus mucosal thickening. Partial bone demineralization of apical periodontitis towards the maxillary sinus floor and an increasing apical periodontitis lesion diameter are two imaging features that significantly increase the thickness of maxillary sinus mucosal thickening. Besides, there is an eighty-two times higher risk of having an odontogenic maxillary sinus mucosal thickening when partly demineralized apical periodontitis towards the maxillary sinus floor is present. Therefore, special attention to maxillary sinus mucosal thickening is required while interpreting CBCT scans, as these may reveal periapical pathology.
